# Using theory to explore facilitators and barriers to delayed prescribing in Australia: a qualitative study using the Theoretical Domains Framework and the Behaviour Change Wheel

**DOI:** 10.1186/s12875-017-0589-1

**Published:** 2017-02-13

**Authors:** Lucy Sargent, Amanda McCullough, Chris Del Mar, John Lowe

**Affiliations:** 1 Centre of Research Excellence in Minimising Antibiotics Resistance for Acute Respiratory Infections (Bond University, Gold Coast), University of the Sunshine Coast, Faculty of Science, Health, Education and Engineering, Sippy Downs, 4556 Australia; 2 Centre for Research in Evidence-Based Practice, Faculty of Health Sciences and Medicine, Bond University, Gold Coast, QLD 4229 Australia; 3 Chair in Population Health Sciences, Faculty of Science, Health, Education and Engineering, University of the Sunshine Coast, Sippy Downs, 4556 Australia

**Keywords:** Delayed prescribing, Acute respiratory infections, Antibiotics, Theoretical Domains Framework, General practitioners, Pharmacists, Patients

## Abstract

**Background:**

Delayed antibiotic prescribing reduces antibiotic use for acute respiratory infections in trials in general practice, but the uptake in clinical practice is low. The aim of the study was to identify facilitators and barriers to general practitioners’ (GPs’) use of delayed prescribing and to gain pharmacists’ and the public’s views about delayed prescribing in Australia.

**Methods:**

This study used the Theoretical Domains Framework and the Behaviour Change Wheel to explore facilitators and barriers to delayed prescribing in Australia. Forty-three semi-structured, face-to-face interviews with general practitioners, pharmacists and patients were conducted. Responses were coded into domains of the Theoretical Domains Framework, and specific criteria from the Behaviour Change Wheel were used to identify which domains were relevant to increasing the use of delayed prescribing by GPs.

**Results:**

The interviews revealed nine key domains that influence GPs’ use of delayed prescribing: knowledge; cognitive and interpersonal skills; memory, attention and decision-making processes; optimism; beliefs about consequences; intentions; goals; emotion; and social influences: GPs knew about delayed prescribing; however, they did not use it consistently, preferring to bring patients back for review and only using it with patients in a highly selective way. Pharmacists would support GPs and the public in delayed prescribing but would fill the prescription if people insisted. The public said they would delay taking their antibiotics if asked by their GP and given the right information on managing symptoms and when to take antibiotics.

**Conclusions:**

Using a theory-driven approach, we identified nine key domains that influence GPs’ willingness to provide a delayed prescription to patients with an acute respiratory infection presenting to general practice. These data can be used to develop a structured intervention to change this behaviour and thus reduce antibiotic use for acute respiratory infections in general practice.

**Electronic supplementary material:**

The online version of this article (doi:10.1186/s12875-017-0589-1) contains supplementary material, which is available to authorized users.

## Background

Antibiotic use contributes to the development of bacterial resistance, [1, 2] which is a significant threat to public health [3]. Acute respiratory infections (ARIs) constitute more than half of all problems treated with antibiotics in general practice [4], but many will resolve without the need for antibiotics [5–7]. Antibiotic resistance appears to be reversible if the selection pressure from antibiotic use is removed [8].

“Delayed prescribing” (also known as “wait-and-see” or “back-up prescription” [9, 10]) is one approach that reduces antibiotic use – 93% of those given an immediate prescription will fill it compared to 32% of those given a delayed script [11]. For patients suffering from cough or common cold, there are no significant differences in duration and severity measures for pain, malaise, fever, cough and rhinorrhoea between patients given a delayed, immediate or no antibiotic prescription. For patients suffering acute otitis media and sore throat, immediate antibiotics do provide slightly quicker relief than those given a delayed prescription but there is an increase in adverse reactions (including nausea, vomiting and diarrhoea) [12, 13]. Patients asked to delay antibiotics were only slightly less satisfied (87%) with care received than those given an immediate antibiotic prescription (92%) [11]. Providing a delayed prescription does not impact re-consultation rates [11] or increase emergency department presentations, [13] is associated with better outcomes [14] and is popular with patients [15].

Delayed prescribing can be used for acute otitis media, acute sore throat/pharyngitis/tonsillitis, the common cold, acute rhinosinusitis and acute cough/bronchitis. Delayed prescribing is the process of a general practitioner (GP) making available an antibiotic prescription during the consultation, but asking patients to delay its use, to see if symptoms will resolve first. Patient concerns and expectations should be addressed when GPs and patients agree to using delayed prescribing. Advice should be given about using the delayed antibiotic if symptoms do not stabilise in accordance with the expected course of the illness or symptoms worsen significantly. Prescriptions can be made available in different ways: patient recontacting the practice subsequently to request it by phone; patients collecting their pre-written prescriptions from the practice after the consultation if their condition worsens; post-dating the prescription; and giving prescriptions to patients while asking them to wait some days before having it dispensed. This last method seems most suitable for the Australian fee-for-service health system. Delayed prescribing has not been implemented in Australian general practice, and local facilitators and barriers to the uptake of this intervention have not been explored. It is the authors belief that delayed prescribing could be one successful strategy Australian GPs could utilise to reduce the overuse of antibiotics.

A growing body of research [17–19] supports the use of psychological theories to identify local facilitators and barriers to changing clinicians’ behaviour [20, 21]. The Behaviour Change Wheel (BCW) is an eight-step guide to designing interventions using a theoretical approach [22]. The first four steps help researchers understand the target behaviour: (1) define the problem in behavioural terms, being specific about the target population and the behaviour itself; (2) select the target behaviour from a list of potential competing behaviours; (3) specify the target behaviour in terms of who needs to do what, when, where, how often and with whom; (4) identify what needs to change in the person or environment in order to achieve the desired behaviour by analysing interview data using the Theoretical Domains Framework (TDF) [23]. The TDF comprises 14 theoretical ‘domains’ representing a range of possible theory-based facilitators and barriers to behaviour change [24].

The primary aim of this study was to use the BCW and the TDF to define delayed prescribing by GPs in behavioural terms, and to identify GPs’ facilitators and barriers to changing this behaviour. The secondary aim was to explore pharmacists’ and the public’s views on delayed prescribing, using the TDF as a framework for analysis.

## Methods

GPs, pharmacists and members of the public completed a single face-to-face, one-on-one, semi-structured interview (February 2014 to July 2015) with the principal researcher (LS, who had previous interviewing experience and no prior relationships with any participant).

### Inclusion and exclusion criteria

Eligible GPs were practising and regularly seeing patients with ARIs. Eligible pharmacists were practising in community pharmacy, and eligible members of the public were those between 18 and 65 years whom accepted the invitation to participate in the research.

### Recruitment strategy

We recruited participants from the Gold Coast and the Sunshine Coast, Queensland, and continued until data saturation was reached. LS transcribed the interviews. Recruitment ceased when all domains had been populated and no new themes within each domain emerged [25].

We recruited GPs practising on the Gold Coast by telephone using snowball sampling. One researcher (CDM) provided a list of 8 GPs to approach for interview. LS telephoned them directly (two agreed). After each interview was completed, the researcher requested a name of another GP to contact. This continued until 5 GPs had been interviewed and no new contacts occurred. GPs on the Sunshine Coast were recruited in person using convenience sampling. Medical practices were identified using an internet search. LS walked into medical practices and requested to see the practice manager. LS extended an invitation to participate which the practice manager distributed via email to all GPs in that practice. GPs contacted the researcher directly by telephone or email. Thirteen GPs agreed to be interviewed. Due to difficulties in recruiting GPs, GPs recruited later in the study (mainly the Sunshine Coast) were offered compensation for the time taken for interview (between $70 and $100 depending on length of interview; figures derived from 2015 Royal Australian College of General Practitioners Medicare Benefits Schedule fee summary) [26]. Only half of the GPs interviewed took the offer of compensation. The researcher arranged an appointment with GPs, who were aware that the consultation was for research, not medical purposes.

We recruited community pharmacists on the Sunshine Coast using convenience sampling and face-to-face discussion with pharmacy managers, who either agreed to be interviewed or allocated a member of staff for interview.

We recruited members of the public (in person) in shopping malls, schools (parents only), universities and coffee shops, and some sitting on park benches, using maximum variation sampling (across gender, age and educational status). LS kept a table to document age, gender and educational status of the public interviewed. Gaps in the table identified the ideal demographic of next participant.

### Setting

Interviews took place in locations convenient for participants: (1) GPs – at home, in general practices and coffee shops; (2) pharmacists – in their pharmacies; (3) the public – at home, outside school gates and in coffee shops. No others were present during interviews.

### Data collection and analysis

**Fig. 1:**
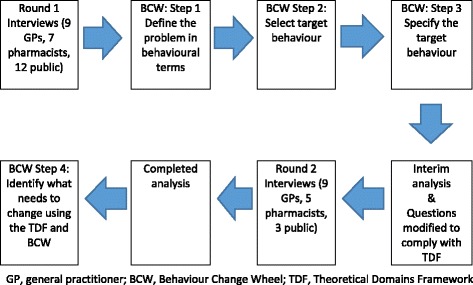
Data collection and analysis process

Figure 1 shows the process of data collection and analysis.

- *Step 1 (Define the problem in behaviour terms)*: We (LS and AMcC) used three questions to define the problem [22]: (1) What behaviour?; (2) Where does the behaviour occur; (3) Who is involved in the behaviour?
- *Step 2 (Select the target behaviour):* We (LS and AMcC) generated a list of all potential GP behaviours relevant to the target behaviour, including influences out of scope (see Additional file 1). To identify the most important target behaviour, we used four criteria [22] and generated a rating system for each criteria: (1) How much of an impact will changing this target behaviour/issue have on outcome? (1 = *highly unlikely*, 2 = *likely* and 3 = *very likely*); (2) How likely it is that the behaviour can be changed? (1 = *yes*, 0 = *no*); (3) How likely it is that the behaviour (or group of behaviours) will have a positive or negative impact on other related behaviours? (1 = *negative* or *no impact*, 2 = *potential impact*, 3 = *positive impact*); (4) How easy will it be to measure the behaviour? (1 = *difficult*, 2 = *possible*, 3 = *easy*). A total score for each behaviour on the list was generated. LS and AMcC used this score along with discussion to reach consensus about which behaviour to target.
- *Step 3 (Specify the target behaviour):* We defined the target GP behaviour from Step 2 using the following questions [22]: (1) Who needs to perform the behaviour? (2) What does the person need to do differently? (3) When will they do it? (4) Where will they do it? (5) How often will they do it? (6) With whom will they do it?
- *Step 4 (Identify what needs to change to achieve target behaviour): *

We collected two rounds of interview data for this step (Fig. 1). Round 1 interview questions explored broad factors influencing GPs’, pharmacists’ and the public’s beliefs on delayed prescribing (see Additional file 2). We did not design this topic guide using the TDF and had not defined the problem in behavioural terms when these interviews were conducted, but did complete steps 1–3 before analysis of Round 1 data took place, allowing us to identify domains not explored that may have been important. We piloted interview questions with one GP, 3 pharmacists and 3 members of the public. No pilot interviews were used in the final data set. No repeat interviews were required. Two GPs requested a copy of the transcripts before analysis. Transcribed interviews were sent via secure email, with a request to reply within 2 weeks if issues were identified; no replies were received. Field notes were made after each interview for use during analysis.

LS recorded and transcribed verbatim all Round 1 interviews. Two researchers (LS and AMcC) coded each transcript independently by assigning each relevant piece of data to one or more TDF domains using NVivo (10 QSR International). All coding was discussed and agreed by the two researchers, one of whom (AMcC) had previous experience using the TDF [27]. From this process, we identified that some domains had not been fully explored (skills, beliefs about capability, optimism, intentions, goals, reinforcement and emotion). We then modified the topic guide (interview questions) to target the domains that required further exploration (see Additional file 3). Two researchers (LS and AMcC) analysed the interviews as previously described for Round 1. In addition, within each category, the researchers (LS and AMcC) identified facilitators and barriers to the target behaviour. The process was iterative and, as new data were added to TDF domains, facilitators and barriers were modified, expanded or re-categorised.

We (all authors) agreed relevant domains (i.e. those directly influencing the target behaviour) by consensus using three questions: (1) What needs to happen for the target behaviour to occur? (2) Is there need for change? (3) Is it relevant?

Table 1 outlines the domains identified as important: Knowledge; Physical, cognitive and interpersonal skills; Memory, attention and decision processes; Optimism; Beliefs about consequences; Intentions; Goals; Emotion and Social influences.

**Table 1:** Step 4 in Behaviour Change Wheel- Identifying what needs to change using the TDF and the BCW

Theoretical Domains	What needs to happen for the target behaviour to occur?	Is there need for change?	Relevance	In/out
Physical skills		no	no	out
Knowledge	Know the correct DP technique	yes	yes	in
Physical, cognitive and interpersonal skills	Physical skills to write a delayed prescription………………………………	no	no	out
	Cognitive skills to conduct delayed prescribing………………….	no	no	out
	Interpersonal skills to reassure patients DP can work ………………	yes	yes	in
Memory, attention and decision processes	Use delayed prescribing and be able to identify the right patients to use it with	yes	yes	in
Behavioural regulation	A method of identifying a delayed script	no	no	out
Professional/social role and identity	GPs need to believe that using delayed prescribing is part of their role	no	no	out
Beliefs about capabilities	GPs need to believe that they can write a delayed prescription	no	no	out
Optimism	Need to believe that delayed prescribing will reduce antibiotic use	yes	yes	in
Beliefs about consequences	GPs need to believe that patients will delay…………………….	yes	Yes	in
	There won’t be adverse consequences if they use delayed prescribing…………………. (i.e. patient won’t die or patients won’t come back)	yes	yes	in
Intentions	GPs have to make a conscious decision to do delayed prescribing	yes	yes	in
Goals	GPs have to want to use delayed prescribing	yes	yes	in
Reinforcement	GPs needs reassurance governing bodies and guidelines are supportive of delayed prescribing	yes	no	out
Emotion	GPs need to feel safe to use delayed prescribing	yes	yes	in
Environmental context and resources	Time with patient	yes	no	
	Resources	yes	no	out
Social Influences	GPs need to trust their patients to be able to follow their advice on delayed prescribing	yes	yes	in

*GP* general practitioner

## Results

Eighteen GPs (8 females, mean age 43 years [range 29–61 years], mean 13 years in practice [range 1–33 years]) with 7 GPs using delayed prescribing, 2 regularly. Two GPs interviewed were married to each other and worked at the same practice but had opposing views on delayed prescribing. Nine pharmacists and three pharmacy assistants (11 female, mean age 36 years [range 24–56 years], mean 10 years in practice [range 1–34 years]) participated. Approximately 35 members of the public were approached for interviews. Fourteen agreed to be interviewed (9 female, mean age 40 years [range 20–61 years], educational status ranged from completion of secondary school to completion of tertiary degrees). One pharmacist withdrew due to time constraints; no GPs or members of the public withdrew. Mean interview time was 29 min (range 13–45 min).

**Fig. 2:**
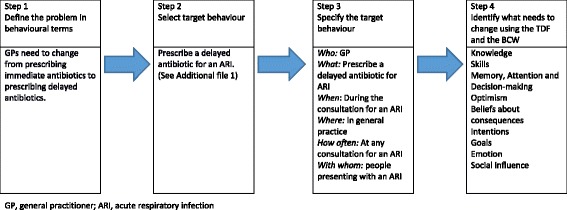
Results of Behaviour Change Wheel process, steps 1–4

Figure 2 illustrates the key results from steps 1 to 4. We defined the problem as the need for GPs to change from prescribing immediate antibiotics to delayed prescription, and specified when and how this should happen. We identified nine relevant domains that influence this behaviour, briefly discussed below. Table 2 provides example quotations from participants that pertain to facilitators and barriers within these domains. The 5 domains identified as irrelevant were behavioural regulation, professional/social role and identity, beliefs about capabilities, reinforcement, and environmental context and resources (Table 1). GPs believed prescribing is part of their role, with delayed prescribing an extension of this role (professional/social role), and that they have the capability to do it (beliefs about capabilities). GPs did not believe systems aimed at monitoring (behavioural regulation), or punishments and rewards (reinforcement), would be appropriate. GPs believed resources (environmental context and resources) are important to help patients manage their symptoms and follow instructions for delayed prescribing; however, they also believed these resources are not relevant to their adopting delayed prescribing.

**Table 2:** Facilitators and barriers to implementing a delayed prescribing intervention based on the Theoretical Domains Framework

Domain	Sub-category	GP quote	Pharmacist quote	Public quote
Knowledge: an awareness of the existence of something				
Facilitators	Knowledge about delayed prescribing	*“It* [delayed prescribing] *is when you give the patient an antibiotic script and ask them to wait until they get bacterial symptoms before filling it.” (GP19)*	*“Is it* [delayed prescribing] *when the GP doesn’t give an antibiotic straight away and the patient needs to return for review?” (Ph14)*	*“The GP said it’s* [sore throat] *going to go like this for the next few days and that’s fine – don’t fill the script but if it gets to this, then go get the script filled or come back and see me.” (Pt03)*
	Knowledge of symptomatic management	*“I usually say simple analgesia” (GP04)*	*“I would suggest medication for symptomatic management…rest, good food, fluids…”(Ph10)*	*“Plenty of rest, fluids, Panadol” (Pt 04)*
	Knowledge that antibiotic use causes antibiotic resistance	*“In my clinical lifetime we are going to be back to people dying of pneumonia and it is likely it we will have nasty resistant bugs that are going to come through and we will have no more antibiotics.” (GP04)*	*“But it’s* [antibiotic resistance] *got really bad now – with all these superbugs”(Ph06)*	*“I know there is more and more resistance to antibiotics and this is becoming an issue, especially if people don’t take their antibiotics properly and then the bugs mutate, and it goes on and on.” (Pt07)*
	Knowledge of when to start antibiotics	*“…but if they* [patients] *know the symptoms on what to look out for, then they can get started on them straight away and see how they go with it” (GP18)*	*“If the GP discusses it* [the illness] *with the patient and lets them know that they don’t have an infection yet and if it does develop in a few days and explains it correctly…” (Ph07)*	*“I probably need to know what to watch out for in case it was getting any worse. Then I would need to know what to do next. So, what’s the point* [I need] *to* [start to] *worry?” (Pt12)*
Barriers	Lack of knowledge about delayed prescribing	*“…it’s more about bringing them* [patients] *back in few days to see the progression of the illness…” (GP02)*	*“I don’t know much* [about delayed prescribing]*” (Ph04)*	*“No, no-one has ever asked me to wait…” (Pt14)*
	Diagnostic uncertainty	*“It’s a hard call as to whether antibiotics are needed often with respiratory tract stuff” (GP16)*	*“…hard to know…we kind of explore it a bit further and suggest they need to go to the doctor for further investigation” (Ph09)*	*“…it’s difficult to know when it* [respiratory infection] *turns” (Pt13)*
Skills: an ability or proficiency acquired through practice				
Facilitators	No physical skills required to do delayed prescribing	*“It’s* [delayed prescribing] *not difficult at all, look, it’s our job to give advice, to educate, to give our opinion. I am a GP and that’s just what I do.” (GP19)*	*“Look, it* delayed prescribing] *shouldn’t be hard – it’s coming down to talk to patients about a prescription, this is what we should be doing all the time.” (Ph11)*	*“No it’s* [delayed prescribing] *just a matter of putting a pill in your mouth and swallowing it, or not.” (Pt15)*
	Interpersonal skills	*“Often getting personal at this point puts it* [informing patients antibiotics are not necessary] *in a different perspective – I am not giving in to you because I don’t like you but if this is my child, or my grandmother, or my mother – this is what I would want to happen.” (GP02)*	*“I think we need to read people well, and sense where the conversation is going.” (Ph14)*	*“My GP has really good people skills. He doesn’t talk to me like I am stupid” (Pt10)*
	Managing expectations (asking patients)	*“Of course – this is a pretty reasonable thing* [asking patients what their expectations are]*…having people coming in here all the time – it is difficult to know what their expectations are” (GP10)*	*“I ask them* [patients] *what we can do next, oh – what they normally do when they are sick like this.” (Ph11)*	*“They* [GPs] *do that these days – ask you what your expectations are” (Pt11)*
Barriers	Managing expectations (not asking patients)	*“No I am not a ‘what are your expectations’ doctor. But after the person brings up ‘oh, I also want this and this…’ which is the third totally separate issue, and I have already spent 15 or 20 min with them, then you do ask yourself – why didn’t I say right at the beginning ‘what are your expectations for today’?” (GP11)*	*“Look, there are obviously expectations, both GP expectations and patient expectations” (Ph04)*	*“I said to him* [the GP] *– you have to understand my side of the situation, I know my child…but he was not prepared to” (Pt02)*
Memory, attention and decision processes: retain information, focus selectively and choose between alternatives				
Facilitators	Identifying patients	*“I use it* [delayed prescribing] *if I think the patient trusts what I am saying and will do what I ask. So, well educated, or just the more savvy person – you can tell, they don’t need to necessarily have a degree” (GP19)*	n/a	*“I am in tune with myself enough to take that on* [decide to commence antibiotics]. *If I still felt bad on day 5, I would take the antibiotics. And I would wait if I was asked.” (Pt08)*
Barriers	Bring patients back for review	*“I tell the patient if they need to, they can come back” (GP12)*	n/a	*“My doctor always says come back and see me…so you don’t feel like you are a bother* [bulk billing practice]”. *(Pt03)*
	Access to medical care	*“Only if…particularly say if I am working in a rural community” (GP13)*	n/a	n/a
	Travel/holiday	*“It will only happen if patients are going overseas or out the country and they ask for it” (GP14)*	n/a	*“This could work* [delayed prescribing]*…if it was a long weekend or I was going travelling, that would be great” (Pt9)*
	Patients demanding antibiotics	*“I can see their agenda is I need to have something, and they aren’t going to feel like they have been cared for by leaving here without anything” (GP18)*	n/a	*“And I said to him* [the GP] *but I don’t want to wait until I am really sick before I get antibiotics.” (Pt02)*
	Previous experience	*“The difficulty too is you sometimes have these people come in and I say ‘oh, it looks all viral’. And then they say: ‘Well, the last time I had this I ended up in hospital with pneumonia’. So what do you do?” (GP07)*	n/a	*“It’s the same with my daughter – she gets sick and then I take time off work, so as a result of the doctor not giving antibiotics early, this is what ends up happening. This is what always happens.” (Pt02)*
	Pain	*“…but if she has a red ear – I would offer an antibiotic. So yeah, if it shortens it by one or two days which is what the studies show – it is 1 or 2 days less of screaming." (GP04)*	n/a	*“…but we [GP and patient] would come to some agreement, like I am the one living with the sick child and listening to him in pain.” (Pt14)*
Optimism: the confidence things happen for the best (includes pessimism)				
Facilitators	Delayed prescribing works	*“I like this approach*– *it* [delayed prescribing] *gives the patient some control in their sickness, isn’t this what we want patients to do? Surely this is a good thing, helping patients take more responsibility and educating them.” (GP19)*	*-*	*“I didn’t need it* [antibiotic] *in the end” (Ph10 speaking as a patient)*
	Implementing an intervention could work	*“I think it* [a delayed prescribing intervention] *would be* [successful]. *Especially with GPs – I think they know that patients like to have some control…as long as they* [patients] *understand what it is and why it* [antibiotic] *is not indicated at this point and when does it become, um, that puts the ball in their court.” (GP18)*	*“If we knew the trial was happening and we knew a prescription was part of it, we should be able to do it. With the understanding if a patient wanted it* [antibiotic] *after the conversation – we would still have to give it.” (Ph11)*	*“I actually quite like this idea* [delayed prescribing] *– you know – I can decide if I am sick enough to need the antibiotic.” (Pt13)*
Barriers	Delayed prescribing will not work	*“No, it* [a delayed prescribing intervention] *will not work. Just they are not reliable – the patients are not reliable. They will go from here and fill the script.” (GP14)*	*-*	*“Oh 95% would walk in* [to a pharmacy] *and get the antibiotic.” (Pt06)*
	Implementing an intervention will be difficult	*“I think it* [a delayed prescribing intervention] *would be hard without more evidence, the obvious question is how many patients go out and fill that prescription immediately.” (GP19)*	*“Only if…if the GP discusses it* [the illness] *with the patient and lets them know that they don’t have an infection yet and if it does develop in a few days and explains it correctly then it might work.” (Ph07)*	*“…if it’s not in their* [the public’s] *face or if they are not suffering or have suffered with an infection that doesn’t respond to medication then how do you make them care?” (Pt14)*
Beliefs about consequences: acceptance of the reality about outcomes of a behaviour in a given situation				
Facilitators	Continued antibiotic use will have serious consequences	*“…we will have nothing to treat the basic infections with…” (GP04)*	*“People are taking several different antibiotics trying to get rid of infections – right down to the clarithromycin’s, and they are still not getting better” (Ph07)*	*“I know there is an increasing issue and there is more and more resistance to antibiotics” (Pt07)*
	Delayed prescribing would reduce antibiotic use	*“…less antibiotics, less resistance, less side effects.” (GP16)*	*“I think it’s* [delayed prescribing] *about them taking medicines safely, isn’t it?” (Ph13)*	*“It’s* [delayed prescribing] *going to make it better all round for antibiotic resistance…” (Pt03)*
	Delayed prescribing would reduce return visits	*“It* [delayed prescribing] *would free up appointments.” (GP18)*	*-*	*“That would save me another trip to the doctor which I like.” (Pt09)*
	Delayed prescribing would provide an educational opportunity	*“…if we take the time to educate patients here, then they learn…they will get better without antibiotics.” (GP19)*	*“…like really, this is a great educational moment” (Ph12)*	*“then maybe that message might get to people” (Pt07)*
	Delayed prescribing would support professional satisfaction	*“…I just know I have done the right thing.” (GP05)*	*“…it* [the reason to adopt delayed prescribing] *is going to have to be a professional incentive, you will know you have stopped them getting an antibiotic when you thought it wasn’t needed”. (Ph10)*	n/a
Barriers	Patients misusing prescriptions	*“Some people are just going to go out and fill the prescription anyway.” (GP19)*	*“It would be interesting to see how many delay.” (Ph12)*	*“Oh 95% would walk in and get the antibiotic.” (Pt06)*
	Missing a serious diagnosis	*“…will I miss a pneumonia or a sepsis in the next few days?” (GP05)*	n/a	*“I would be worried of it* [the illness] *getting worse…” (Pt15)*
	Losing business	*“…so losing patients” (GP05)*	*“We might actually lose business because we wouldn’t fill a prescription.” (Ph11)*	n/a
	Antibiotic stewardship is not my problem	*“So, the enormous prescribing going on to every pregnant sheep or cow” (GP04)*	*“…people don’t see it* [antibiotic resistance] *as their problem.” (Ph07)*	*“I just don’t know how you could make it* [antibiotic resistance] *more real for people” (Pt14)*
	Taking longer	*“This is a big factor* [time] *in general practice because if you are going to adequately explain conditions and why you are going to do what you do and do it appropriately – it takes longer.” (GP07)*	*“It* [delayed prescribing]) *can go really well if the doctors spend time with the patients” (Ph07)*	*“I must have come across a doctor at some stage that explained it* [delayed prescribing] *to me, someone who actually took the time to discuss it” (Pt03)*
Intentions: A conscious decision to perform a behaviour or a resolve to act in a certain way				
Facilitators	Intends to use delayed prescribing	*“No – I just do it* [delayed prescribing] *really* [laughing]. *I don’t consciously think oh I must do that now. I just do it.” (GP16)*	*“Yes – it’s* [talking to patients about their prescriptions] *just one of the things I do. It’s part of a lot of things I just do.” (Ph10)*	*“And I am happy to see the doc again or if, like in your story, wait and see if it gets better or worse. I reckon I could do that.” (Pt11)*
Barriers	No intention to use delayed prescribing	*“But it* [delayed prescribing] *doesn’t come into my mind to use.” (GP12)*	n/a	n/a
Goals: mental representations of outcomes that an individual wants to achieve				
Facilitators	To use delayed prescribing	*“I want to use delayed prescribing a lot if the patient can make an informed decision about their own care.” (GP19)*	n/a	*“If he asked me to do it* [delayed prescribing] *– assuming he writes out everything I need to do and when to do it, I don’t think it would be a problem, it would just be about following instructions.” (Pt15)*
	To feel better	*“They want to be better yesterday and tomorrow is far too long” (GP03)*	*“Generally I think they just want to feel better now…” (Ph06)*	*“And you know, if he* [GP] *said; ‘here, take this instead this would make you feel better’ – then I would probably take that recommendation over the antibiotics.” (Pt13)*
Barriers		*-*	*-*	*-*
Emotions: a complex reaction by which an individual attempts to deal with a significant event				
Facilitators	Reducing anxiety	*“And also I think it* [delayed prescribing] *decreases the anxiety surrounding things like flu-like symptoms because people do feel awful, and will keep coming back but if you have explained it to them and you have given them the management plan…” (GP18)*	*-*	*“Then I would know what to do next – now I need to go to the Emergency Room, or now I need to…whatever…” (Pt12)*
Barriers	Significant event	*“I couldn’t find anything wrong with the child and said it was an URTI and I won’t prescribe antibiotics because I was being really good. Of course – the child was soon having a lumbar puncture and um, having an encephalitis two minutes later, and I was: why didn’t I prescribe antibiotics? Those ones will always stay with you.” (GP04)*	n/a	*“But I thought he* [child] *had a relapse. I just didn’t know what to do – should I have done this or should I have done that…” (Pt14)*
Social influences: the interpersonal processes that can cause individuals to change their thoughts, feelings or behaviours				
Facilitators	Some patients do not want antibiotics	*“Some people are like ‘I don’t want to take anything, I just want to see how it goes. I want to be all natural’.” (GP09)*	*“Most people don’t want medication these days – they want the vitamins and the zinc! It’s such a better way to do ‘sick’, well, I think anyway.” (Ph12)*	*“I am so vocal that I don’t want antibiotics, so they don’t feel the need to do that with me.” (Pt03)*
	Trust in patients	*“Then they* [the patient] *learn for the rest of their lives – they will get better without antibiotics but not just that – they also learn that we have trusted them and it all worked out well.” (GP19)*	*-*	*“They* [GP] *are kind of going well, I am trusting, that would mean they would be trusting me as a mum, to know when my child was getting worse or better. And that I can make that judgement call myself” (Pt04)*
	Relationships with regular patients make it easier to refuse antibiotics	*“Most patients that are regular that we see all the time, they are more likely to know what we will do and again if they know you reasonably well, it is easier to get them not to take things – so if they haven’t got a relationship with you, you are more likely to prescribe.” (GP04)*	*“…mainly because we have regular customers who believe what I say. We do have that relationship. They know I have been right before and I am not making things up or trying to sell them stuff” (Ph07)*	*“I wouldn’t have a problem at all* [if my GP asked me to wait]*, I have a reasonably good relationship with my GP, I don’t always agree with what he says but… then if he asked me to wait, that would be fine with me.” (Pt14)*
	Some patients just want reassurance	*“'You are doing it all ‘correctly' and then they* [patients] *feel reassured and maybe that has a bit of a placebo effect that they are speaking to the doctor and he has said everything is going to be OK” (GP05)*	-	*“just going to the doctor and him telling me I am going to be OK, if he said I didn’t need antibiotics then I would be fine with this, really, I don’t mind being told not to take tablets or to wait for that matter” (Pt13)*
	GPs don’t feel pressured by patients who demand antibiotics	*“Well, I just I guess I tend to be a bit stubborn at times. If they really get up my nose, then I explain as nicely as possible all the reasons that I ah, believe it’s viral. But I will dig my heels in if they really insist” (GP11)*	n/a	*“Another doctor, a younger doctor – he works at the hospital but he also has rooms at the surgery, he wouldn’t give me an antibiotic. He just flatly refused.” (Pt02)*
	GPs are a trusted profession	*“A position of trust I think. Being a doctor. Patients listen to you.” (GP15)*	*“…because not only are you getting the information from the doctor who you trust…” (Ph08)*	*“I do think GPs are very trusted people.” (Pt09)*
Barriers	Expectations	*“The common cold is very very common of course and people come in expecting to have antibiotics.” (GP14)*	*“Do you think it is because the patient goes to see the doctor expecting something?” (Ph09)*	*“…in my generation we were brought up to believe that antibiotics fix everything.” (Pt10)*
	Lack of trust in patients	*“Oh – you see – look, I don’t trust my patients enough for delayed prescribing.” (GP17)*	n/a	n/a
	Social factors	*“A couple things* [influence my prescribing] *– what time of the week it is – like if it’s towards the end of the week – I tend to do more of the delayed prescribing – because they won’t have access to a doc over the weekend. Travel medicine. Cost is a big thing – some people are on a pension and they don’t have a lot of money, co-morbidities – smoking, diabetes, asthma – I tend to prescribe a little bit more.” (GP05)*	*“…well, as I said if we are busy and they* [the patient] *are sick or not receptive.” (Ph13)*	*“When she* [mother] *goes to the doctor for a cold, expecting antibiotics and even when I say – ‘mum these don’t work’, when she asks, he still just gives them to her. I think, for her, it’s to keep her quiet – to send her away, give her a course of antibiotics.” (Pt10)*
	Giving up control	*“Some GPs have this idea that it’s better that you do it* (prescribe antibiotics), *and then at least you are in control of the situation” (GP07)*	n/a	*“If the GP said ‘Here is a prescription for antibiotics’ I would take them.” (Pt12)*

‘-’, Not reported; *n/a* not applicable

### Knowledge

All GPs had heard of delayed prescribing but did not use it correctly or only used it selectively. GPs knew that delayed prescribing involves giving an antibiotic prescription to the patient with instructions on how long to wait and when to take the antibiotics if symptoms worsen.

“…*if you are no better in a couple of days, fill the script. Um, so I am thinking how often I do it, um, I do do that occasionally, especially if it is near the weekend or something and they can’t come back to see me.”* (GP16)

All pharmacists were knowledgeable about management of ARI symptoms. The public reported needing to know when to start antibiotics (if given a delayed prescription) and how to treat their symptoms.

*“So I would need to know when to take the antibiotic, so when is the cold getting worse, how long this takes, the expected length of time for the cough and how it would impact my breathing.”* (Pt15)

All participants knew antibiotic use caused antibiotic resistance.

### Skills

GPs considered they did not need any additional *physical* skills to conduct delayed prescribing. GPs reported using *interpersonal* skills to explore patient expectations regarding antibiotic prescription, although they seldom explicitly ask patients about their expectations.

“[I do not ask them about their expectations]… *in such a straight forward way – like, I normally ask them what they have been doing to cope before they come to me for help.”* (GP19)

All pharmacists reported using interpersonal skills in their interactions with the public to identify customers’ needs. Pharmacists did not believe they need additional skills to conduct delayed prescribing because talking to the public about prescriptions is a skill they use frequently.

*“…we are really big on coming down and talking to everyone about their medication, this would be another thing that we already do.”* (Ph10)

The public didn’t think they need any skills for delaying taking an antibiotic.

### Memory, attention and decision-making

GPs who said they use delayed prescribing use it in highly specific situations: for patients whom they believe are well educated and sensible, and whom they trust will delay filling the prescription. Further, they identified that the only reasons they would use delayed prescribing are if patients have difficulties accessing a GP, are going on holiday, are suffering from otitis media, or demand antibiotics. Some GPs stated they would never use delayed prescribing because they prefer to bring patients back for review.

*“…if they perceive the condition isn’t resolving and to come back if they want.”* (GP10)

Pharmacists had no comment on this domain. The public stated they would wait for worsening symptoms before taking antibiotics but, if a previous infection had progressed to a severe one or they were in pain, they would be more likely to want to take antibiotics immediately.

### Optimism (and pessimism)

The minority of GPs who said they already use delayed prescribing regularly were optimistic about using this strategy. Most GPs believed that it would be difficult to implement delayed prescribing: they do not trust patients to delay and do not believe there is enough evidence to refute this perception. Pharmacists and the public were optimistic about using delayed prescribing.

*“Some people are just going to go out and fill the prescription anyway.” (GP19)*

*“I like the idea that I might be able to make my own decision about when to take antibiotics, this doesn’t frighten me at all”* (Pt13)

### Beliefs about consequences

Participants (GPs, pharmacists and the public) believed antibiotic resistance is a serious issue influenced by antibiotic use. They identified advantages of delayed prescribing as reduced antibiotic use and resistance, providing an opportunity for patient education, and giving professional satisfaction.

“*I think it would be good – it would work, education would be for the people using it”* (GP16)

“*Um, I use it as it means less reviews, less people coming in, I guess giving patients control over their condition.”* (GP09)

*“Um, I guess it makes me feel I am just playing a small part in reducing antibiotic resistance.”* (GP15)

But they were also concerned that the public would misuse the delayed prescription by either using it immediately or keeping it for a subsequent infection. GPs were specifically concerned about missing serious infections, that delayed prescribing could be more time-consuming and that it could potentially lose them business.

“…*but it is easier to give the script because the patient leaves the room immediately. So most of that is time pressure.”* (GP06)

### Intentions

GPs who regularly use delayed prescribing said they do not have to consciously think about it – they believe it is an effective strategy and intend to use it as often as they can. GPs who use delayed prescribing selectively said they need to think carefully if patients fit certain criteria. Most GPs stated they have no intention of using delayed prescribing because they can bring patients back for review. All pharmacists stated that, if they have the time, they intend to talk to patients who request an antibiotic prescription be filled. The public said they intend to do what the GP asks of them.

### Goals

Most GPs did not mention that they had a goal of using delayed prescribing. One GP expressed a strong goal of using delayed prescribing if patients were able to make an informed decision on their care. All groups identified the main goal of sick patients is to feel better.

*“If I was bedridden then I would take anything to get better.”* (Pt08)

### Emotion

This domain overlaps with the ‘beliefs about consequences’ domain – GPs and the public expressed a fear of missing a serious diagnosis - one GP stated he is a regular prescriber of antibiotics because he had had some patients experience severe complications from not starting antibiotics sooner. GPs believe delayed prescribing reduces patient anxiety about disease progression because they have a management plan in place.

### Social influences

Several conflicting social influences were evident. These influences centre on perceived expectations of antibiotic treatment and on trust between clinicians and patients. All GPs said they do not feel pressured when patients demand antibiotics, but they reported mixed views on whether patients expect antibiotics. The time and day of the week, patients’ social situation (e.g. getting back to work, financial status, age and comorbidities) and an existing relationship with a patient influence their antibiotic prescribing. GPs who use delayed prescribing reported the practice encourages trust between GPs and patients. Other GPs reported they don’t trust their patients; however, they contradicted this statement by saying they believe that their patients listen to them.

“*A position of trust I think. Being a doctor. Patients listen to you*.” (GP15)

“*Oh – you see – look, I don’t trust my patients enough for delayed prescribing*.” (GP17)

“*… if we take the time to educate patients here, when they are sitting in the chair, they go home, they don’t use the antibiotic and then they get better, if we are talking about the best case scenario, then they learn for the rest of their lives – they will get better without antibiotics but not just that – they also learn that we have trusted them and it all worked out well*.” (GP19)

Pharmacists acknowledged many people do not want antibiotics to treat their illness and would rather have natural remedies; however, pharmacists also believe some people go to the GP expecting antibiotics. Many members of the public stated they do not want antibiotics if they don’t need them; all identified good relationships with both GPs and pharmacists as important because they often want reassurance they are doing the right thing. The public reported trusting GPs, saying they would do what the GP asks of them and that delayed prescribing indicates that the GP trusts them to make decisions about their own health.

*“Even if it was also just re-assurance that I knew what I was talking about – and then he wouldn’t need to hand over that antibiotic prescription straight over, and then I wouldn’t need to start them on it straight away.”* (Pt04)

## Discussion

### Main findings

To implement delayed prescribing in Australian general practice, GPs need to change from immediate antibiotic prescribing to delayed prescribing and educate patients about how to manage ARI symptoms and when to start antibiotics. This education can take place during any consultation with a person presenting with an ARI in a general practice setting. Nine TDF domains influence this behaviour: knowledge; skills; memory, attention and decision-making; optimism; beliefs about consequences; intentions; goals; emotion; and social influences. Pharmacists and members of the public are broadly supportive of using delayed prescribing; whilst most had not heard of delayed prescribing, they were optimistic that it could be a useful strategy to reduce antibiotic use.

Although effective, uptake of delayed prescribing by general practitioners (GPs) appears to be low: one study found that 15% of patients prescribed antibiotics in the past year had been given the option of a delayed prescription [16]. These findings may be helpful to researchers trying to implement an intervention to support delayed prescribing in other fee-for-service environments.

### Strengths and limitations

The main strengths of this study are the systematic and structured approach to defining and specifying, in behavioral terms, the problem of implementing delayed prescribing, and the use of a theory-driven approach to identify facilitators and barriers to changing this behaviour. Interviewing pharmacists and members of the public ensured that views from these two key groups of stakeholder can be used to inform the development of a future intervention.

The main limitation of this study is that the TDF was not used to design the topic guide for Round 1 interviews as the BCW work book was not published and the TDF was not identified as a method for analysis until after the interviews had commenced. However, the TDF was used for the interim analysis and to identify gaps in the emerging data that were subsequently addressed by modifying the interview questions. More than one-third of GPs in this sample reported using delayed prescribing – higher than the uptake rates estimated earlier, [16] which could indicate selection bias, in that GPs who were supportive of delayed prescribing were more likely to agree to be interviewed. There could also have been social desirability bias in all interviews where participants answered the questions in a manner that would be viewed as favourable by the researcher. Snowball sampling of GPs on the Gold Coast and convenience sampling of participants on the Sunshine Coast may also have introduced selection bias. Further, most of the pharmacists interviewed were female. Participants were encouraged to arrange interviews at a time and place most convenient to them, with most interviews of GPs and pharmacists occurring at workplaces. This factor may have affected participants’ responses due to time pressure, and may have led to participants’ feeling less able to disclose certain information. The decision about whether to prescribe antibiotics for patients with ARIs is potentially a sensitive issue. Hence, GPs may be reticent to divulge overprescribing – only one GP admitted to being a high prescriber. We did not collect data on GPs’ current rate of antibiotic prescription.

### Comparison with existing literature

Similar to findings in other qualitative studies of delayed prescribing, [28–33], we found that Australian GPs were concerned about the misuse of prescriptions by patients, diagnostic uncertainty and patient hoarding of medication. We also found that Australian GPs were often highly selective regarding the patients for which they used delayed prescribing. Some of these concerns are not consistent with the evidence: 32% of patients fill a delayed prescription whilst 93% fill an immediate prescription [11, 20, 34, 35]. Hoarding could be a valid concern. When asked about repeat prescriptions (for any indication), one-third of Australian respondents stated they would keep prescriptions for future use [36]. To our knowledge, this study is the first to explore facilitators and barriers to delayed prescribing using the TDF and the BCW. However, this approach has been used successfully for developing other interventions [27, 37–39].

### Implications for clinicians and policy makers

There is adequate evidence that delayed prescribing reduces antibiotic use, [11] and the concept has been previously introduced into the Australian GP environment [40]. However, our interviews indicate the practice is not consistently used, with many GPs expressing concerns about its implementation. GPs can use the results of this study to identify their individual facilitators and barriers to using this strategy in their own practice. Policymakers and researchers will need to consider the facilitators and barriers to delayed prescribing identified in this study before designing an acceptable implementation intervention.

### Unanswered questions and future research

We have identified theory-derived facilitators and barriers to the implementation of delayed prescribing in Australian general practice. The next step is to use the BCW to identify which behaviour-change techniques could be used to target the TDF domains that influence the use of delayed prescribing by Australian GPs, and then to test the feasibility of this theory-based intervention in Australian general practice.

## Conclusion

Using a theory-driven approach, we have identified nine key domains that influence Australian GPs’ willingness to provide a delayed prescription to patients with an ARI presenting to general practice. These data can be used to develop a structured intervention to change GPs’ behaviour and thus reduce antibiotic use in Australian general practice.

## Additional files

Additional file 1: Step 2 in Behaviour Change Wheel. (DOCX 15 kb)
Additional file 2: Topic Guide for Round 1 interview questions. (DOCX 14 kb)
Additional file 3: Topic guide for Round 2 interview questions (modified: Step 4a in Behaviour Change Wheel). (DOCX 20 kb)

## Abbreviations

ARI: Acute respiratory infection
BCW: Behaviour Change Wheel
GP: General practitioner
TDF: Theoretical Domains Framework
