# Supplementary material for: Using theory to explore facilitators and barriers to delayed prescribing in Australia: a qualitative study using the Theoretical Domains Framework and the Behaviour Change Wheel
Source: BMC Fam Pract. 2017 Feb 13;18:20. doi: 10.1186/s12875-017-0589-1 (PMC5307801; doi:10.1186/s12875-017-0589-1)
Supplement: Additional file 1: — Step 2 in Behaviour Change Wheel. (DOCX 15 kb) [file 12875_2017_589_MOESM1_ESM.docx]

**Additional file 1: Step 2 in Behaviour Change Wheel – List of potential target behaviours**

**The behaviour we want to change is for GPs to prescribe a delayed antibiotic for an acute respiratory infection.**

Questions used to prioritise behaviours:

C1 = how much of an impact changing this target behaviour/issue will have on outcome (rated as 1 if *highly unlikely*, 2 if *likely* and 3 if *very likely*)

C2 = how likely it is that the behaviour can be changed; when considering likelihood of change being achieved, think about the capability, opportunity and motivation to change of those performing the behaviour (1 indicates *yes* and 0 indicates *no*)

C3 = how likely it is that the behaviour (or group of behaviours) will have a positive or negative impact on other related behaviours (1 is a *negative or no impact*; 2 is a *potential impact* and 3 is a *positive impact*)

C4 = how easy it will be to measure the behaviour (1 is *difficult*, 2 is *possible* and 3 is *easy*)

| **Targeted behaviour** | **C1** | **C2** | **C3** | **C4** | **Total** |
| --- | --- | --- | --- | --- | --- |
| **GP practice** |  |  |  |  |  |
| Prescribing an immediate antibiotic | n/a | n/a | n/a | n/a | - |
| Prescribing a delayed antibiotic for an acute respiratory infection | 3 | 1 | 3 | 2 | 9 ✔ |
| Not prescribing an antibiotic | 3 | 1 | 3 | 2 | 9 |
| Prescribing an alternative | 3 | 1 | 3 | 2 | 9 |
|  |  |  |  |  |  |
| **GP environment** |  |  |  |  |  |
| Providing GP with educational resources to support DP | 3 | 1 | 3 | 1 | 8 |
| Providing access to information on DP | 3 | 1 | 3 | 1 | 8 |
| Providing a system that allows post-dated prescriptions | 3 | 0 | 1 | 1 | 5 |
| Providing a process for delayed scripts to be collected | 3 | 1 | 1 | 1 | 6 |
| Providing a system allowing expiry dates to be added to prescriptions | 2 | 0 | 1 | 1 | 4 |
| Including DP as an audit item for CPD points | 2 | 0 | 2 | 1 | 5 |
| Creating and sharing a DP policy for GP practices | 1 | 1 | 1 | 3 | 6 |
| **Legislation** |  |  |  |  |  |
| Providing longer consultation times | 2 | 0 | 2 | 1 | 5 |
| Changing funding system | 3 | 0 | 3 | 1 | 7 |
| Adding DP to prescribing guidelines | 1 | 0 | 1 | 1 | 3 |

C, Criteria; GP, general practitioner; n/a, not applicable; DP, delayed prescribing; CPD, continuous professional development
