# Supplementary material for: Using theory to explore facilitators and barriers to delayed prescribing in Australia: a qualitative study using the Theoretical Domains Framework and the Behaviour Change Wheel
Source: BMC Fam Pract. 2017 Feb 13;18:20. doi: 10.1186/s12875-017-0589-1 (PMC5307801; doi:10.1186/s12875-017-0589-1)
Supplement: Additional file 2: — Topic Guide for Round 1 interview questions. (DOCX 14 kb) [file 12875_2017_589_MOESM2_ESM.docx]

**Additional file 2: Topic Guide – Round 1 interview questions**

| **Theme** | **GPs** | **Pharmacists** | **Public** |
| --- | --- | --- | --- |
| Delayed prescribing | Do you know about delayed prescribing?    Do you use delayed prescribing? Why/why not?  How often do you use delayed prescribing?  What do you think would help you use delayed prescribing?  Which patients would you use delayed prescribing for?  What do you think will happen if you use delayed prescribing more?  What clinical reasons would you use delayed prescribing for?  Can you tell me about a specific consultation in which you gave a delayed prescription? (Or no prescribing)?  If you were going to use delayed prescribing in a consult with a patient who was expecting antibiotics, what would you need to facilitate this? | What is your understanding of delayed prescribing?  How did you manage the last customer who presented with respiratory symptoms?  What concerns do you have if you were going to ask customers to wait and see if they get better before filling their antibiotic prescription? | Tell me about a time the GP asked you to wait before taking your antibiotics?  What has been your experience when GPs haven’t given you an antibiotic prescription when you were expecting one?  What else would you like if you weren’t given an antibiotic prescription? |
| Antibiotic use | If you don’t give an antibiotic when it is expected, do you give the patient anything else?  When do you discuss antibiotic resistance with your patients as a reason for not giving an antibiotic prescription?  What worries you the most if you don’t prescribe an antibiotic? | Do you ever explicitly discuss antibiotic resistance with your customers as a reason for not taking antibiotics?  Do you see yourself in a role that needs to raise awareness of judicious use of antibiotics? | What has been your experience when GPs haven’t given you an antibiotic prescription when you were expecting one?  If you aren’t given an antibiotic when it is expected, what would you like instead?  Does your GP ever explicitly discuss antibiotic resistance with you when giving you an antibiotic prescription?  What worries you the most if you don’t get a prescription when you are expecting one? |
| Influences | Do you ask patients about their expectations during consultations?  Do you see other health professionals having a role in raising awareness of judicious use of antibiotics?  How have your ARI antibiotic prescribing habits changed over the last 5 years?  How do you see GPs’ antibiotic prescribing habits changing in the future? | What influences your discussions with customers?  How has this changed over the years?  Do you talk to customers about their expectations during discussions?  How do you see GPs’ antibiotic prescribing habits changing in the future? | Tell me about the last time you had a really bad cough, cold or sore throat.  What information did your GP give you about your condition?  How has the way your GP treated you for cough and colds changed?  Do you see other health professionals having a role in raising awareness of antibiotic resistance? |

GP, general practitioner; ARI, acute respiratory infection
