# Supplementary material for: Using theory to explore facilitators and barriers to delayed prescribing in Australia: a qualitative study using the Theoretical Domains Framework and the Behaviour Change Wheel
Source: BMC Fam Pract. 2017 Feb 13;18:20. doi: 10.1186/s12875-017-0589-1 (PMC5307801; doi:10.1186/s12875-017-0589-1)
Supplement: Additional file 3: — Topic guide for Round 2 interview questions (modified: Step 4a in Behaviour Change Wheel). (DOCX 20 kb) [file 12875_2017_589_MOESM3_ESM.docx]

**Additional file 3: Step 4a in Behaviour Change Wheel – Topic guide for Round 2 interviews (modified using the Theoretical Domains Framework)**

| **Domain** | **Definitions** | **GPs** | **Pharmacists** | **Public** |
| --- | --- | --- | --- | --- |
| Knowledge | An awareness of the existence of something; includes knowledge of condition or scientific rational, procedural knowledge and knowledge of task environment. | Do you know about delayed prescribing?  How do you treat an acute respiratory infection?  Do you ever explicitly discuss antibiotic resistance with your patients as a reason for not taking antibiotics? | Do you know about delayed prescribing?  What do you know about antibiotic resistance?  What would you suggest for a customer with an acute respiratory infection? | Have you ever had a GP give you an antibiotic prescription but asked you not to fill it until you get worse?  Do you know about antibiotic resistance?  What do you normally do when you have a respiratory infection?  If a doctor examined you and said you didn’t need an antibiotic but gave you a prescription for later in case you got worse, what sort of information would you need to feel safe? |
| Skills | An ability or proficiency acquired through practice; includes skills, skills development, competence, ability, interpersonal skills, practice and skill assessment. | Do you ask patients what their expectations are?  What skills do you need to talk to patients about delaying their prescriptions? | What skills do you need to talk to customers about their prescriptions? | What skills do you think you need if you were asked not to fill your antibiotic prescription? |
| Memory, attention and decision processes | The ability to retain information, focus selectively on aspects of the environment and choose between two or more alternatives; includes memory, attention, attention control, decision making, cognitive overload/tiredness. | Is delayed prescribing something you usually do?  How do you decide when to use delayed prescribing? | Is talking to a customer about their antibiotic prescription something you usually do? | Do you usually want to take an antibiotic for a respiratory infection?  How do you decide what to treat your respiratory infection with? |
| Behavioural regulation | Anything aimed at managing or changing objectively observed or measured actions; includes self-monitoring, breaking habit and action planning. | Is there anything that regulates your prescribing habits?  Where do you find information about how to prescribe?  What resources do you use when talking to patients about antibiotics?  What do you think you need to be able to do delayed prescribing?  Do you give patients anything else besides an antibiotic? | What resources do you use when talking to customers about antibiotics?  Who should be talking to customers about antibiotics?  What do you think you need to be able to do delayed prescribing? | Were you given anything, besides a prescription, when you went to the doctor about your cough/cold/sore throat? |
| Social/ professional role | A coherent set of behaviours and displayed qualities of an individual in a social or work setting; includes professional identity, professional role, social identity, identity, professional boundaries, professional confidence, group identity, leadership and organisational commitment. | Do other health professionals have a role in supporting GPs to do delayed prescribing?  Do you talk to patients about antibiotic resistance?  Is using delayed prescribing compatible or in conflict with your professional standards?  Who should be talking to patients about antibiotics? | Do you see yourself in a role that needs to raise awareness of judicious use of antibiotics?  Do you talk to customers about antibiotic resistance?  Is talking to customers about their prescription compatible or in conflict with your professional standards? | Has a health professional ever talked to you about antibiotic resistance?  Who do you think needs to play a role in raising antibiotic resistance awareness?  How would you feel about a pharmacist asking you to wait before you filled the antibiotic prescription? |
| Beliefs about capabilities | Acceptance of the truth, reality, or validity about an ability, talent, or facility that a person can put to constructive use; includes self-confidence, perceived competence, self-efficacy, perceived behavioural control, beliefs, self-esteem, empowerment and professional confidence. | How difficult is it to not prescribe when patients want antibiotics?  How difficult is it to do delayed prescribing?  How easy is it to do delayed prescribing?  How difficult would you find it to talk to patients about not filling the antibiotic prescription? | How difficult is it to talk to customers about their prescriptions?  How easy is it to talk to customers about their prescriptions?  How difficult would you find it to talk to customers about not filling the antibiotic prescription? | How difficult would it be not to fill the antibiotic prescription?  How easy would it be not to fill the antibiotic prescription? |
| Optimism | The confidence that things will happen for the best or that desired goals will be attained; includes optimism, pessimism, unrealistic expectations and identity. | How confident are you that delayed prescribing could be a successful strategy? | How confident are you that an intervention supporting pharmacists to have these conversations with customers will be successful? | How confident are you that delayed prescribing could be successful? |
| Beliefs about consequences | Acceptance of the truth, reality, or validity about outcomes of a behaviour in a given situation; includes beliefs, outcome expectancies, and characteristics of outcome expectancies, anticipated regret and consequences. | What consequences do you foresee if we continue to use antibiotics like we are?  What advantages can you see if you used delayed prescribing?  What disadvantages can you see of using delayed prescribing? | What consequences do you foresee if we continue to use antibiotics like we are?  What advantages can you see if you used delayed prescribing?  What disadvantages can you see of using delayed prescribing?  What do you think will happen if you have conversations with customers about delaying their antibiotic prescription? | What advantages can you see if you were asked to use delayed prescribing?  What disadvantages can you see if you were asked to use delayed prescribing?  What do you think would happen if you didn’t take the antibiotic? |
| Intentions | A conscious decision to perform a behaviour or a resolve to act in a certain way; includes stability of intentions, stages of change model, trans-theoretical model and stages of change. | Have you made a conscious decision to use delayed prescribing? | Do you have to make a conscious decision to approach customers about their antibiotic prescriptions? | Do you intend to take antibiotics for a respiratory infection? |
| Goals | Mental representations of outcomes or end stages that an individual wants to achieve. | How much do you want to do delayed prescribing? | How much do you want to have this conversation with customers? | How much do you not want to take antibiotics? |
| Reinforcement | Increasing the probability of a response by arranging a dependent relationship, or contingency, between the response and a given stimulus; includes rewards, incentives, punishments, consequents, reinforcement, contingencies, and sanctions. | Are there incentives to do delayed prescribing? | Are there incentives to have conversations with customers about their antibiotics? | What incentives can you think of for not taking antibiotics or waiting before you take them? |
| Emotion | A complex reaction pattern involving experiential, behavioural and physiological elements, by which the individual attempts to deal with a personally significant matter or event; includes fear, anxiety, affect, stress, depression, positive or negative affect and burn-out. | Does the idea of using delayed prescribing evoke an emotional response? | Does the suggestion of asking customers about their antibiotic prescription evoke an emotional response? | Can you think of an emotional response that you might have when asked to wait before taking antibiotics? |
| Environmental context and resources | Any circumstance of a person’s situation or environment that discourages or encourages the development of skills and abilities, independence, social competence and adaptive behaviours. | To what extent do you think your environment helps you to do delayed prescribing with a patient?  To what extend do you think your environment hinders you to do delayed prescribing with a patient? | To what extent do you think your environment helps you to talk to a customer about their prescription?  To what extent do you think your environment hinders you to talk to a customer about their prescription? | To what extent do you think the environment helps you to delay filling a prescription?  To what extent do you think the environment hinders you to delay filling a prescription? |
| Social influences | Those interpersonal processes that can cause individuals to change their thoughts, feelings and behaviours; includes social pressure, social norms, group conformity, social comparisons, group norms, social support, power, intergroup conflict, alienation, group identity, modelling. | To what extent do patients’ social influences help you to do delayed prescribing?  To what extent do patients’ social influences hinder you to do delayed prescribing?  To what extent could pharmacists help you to delay filling your prescription?  To what extent could pharmacists hinder you to delay filling your prescription?  To what extent do other health professionals hinder you to do delayed prescribing? | To what extent do customers and their social influences help you to talk to them about their prescriptions?  To what extent do customers and their social influences hinder you to talk to them about their prescriptions?  To what extent do you think you could help the GP do delayed prescribing? | Has anything influenced you in how you care for your respiratory infection? |
